# Engineered spermidine-secreting *Saccharomyces boulardii* enhances olfactory memory in *Drosophila melanogaster*

**DOI:** 10.3389/fnins.2025.1628160

**Published:** 2025-10-31

**Authors:** Florance Parweez, Roger Palou, Ruizhen Li, Lanna Kadhim, Heath MacMillan, Mike Tyers, X. Johné Liu

**Affiliations:** ^1^Inflammation and Chronic Disease Program, Ottawa Hospital Research Institute, Ottawa, ON, Canada; ^2^Department of Biochemistry, Microbiology and Immunology, University of Ottawa, Ottawa, ON, Canada; ^3^Program in Molecular Medicine, The Hospital for Sick Children Research Institute, Toronto, ON, Canada; ^4^Department of Biology and Institute of Biochemistry, Carleton University, Ottawa, ON, Canada; ^5^Department of Molecular Genetics, University of Toronto, Toronto, ON, Canada; ^6^Department of Obstetrics and Gynecology, University of Ottawa, Ottawa, ON, Canada

**Keywords:** *S. boulardii*, spermidine, *D. melanogaster*, short-term memory, gastrointestinal tract, gut-brain-axis, diuretic hormone Dh31

## Abstract

**Introduction:**

The polyamines putrescine, spermidine, and spermine are ubiquitous metabolites synthesized in all cells. The intracellular levels of polyamines, especially spermidine, decrease in aging. Oral spermidine supplementation has been reported to alleviate aspects of aging-related disease in animal models, including decline in learning and memory. The diverse health benefits of spermidine supplementation, often at doses that do not significantly alter spermidine levels of target organs, suggests that exogenous spermidine may have a common site of action, the gastrointestinal (GI) tract.

**Methods:**

To directly deliver spermidine to the GI tract with minimum impact on the global spermidine levels, we engineered the probiotic yeast *Sacchromyces boulardii* (*Sb*) to overproduce and secrete spermidine. We tested the effects of a spermidine-producing yeast strain (Sb576) on aging-associated learning and memory decline in an olfactory classical conditioning assay in *Drosophila melanogaster*.

**Results:**

Feeding of newly eclosed flies of the wild-type (w^1118^) strain for 30 days with food supplemented with live Sb576, but not live wild-type *Sb* (SbWT) or free spermidine, reduced aging-associated short-term memory (STM) decline. Notably, Sb576 supplementation, but not SbWT or spermidine supplementation, of either young flies or old flies for only three days also enhanced STM without affecting locomotive ability. Furthermore, we showed that Sb576 supplementation also significantly reduced aging-associated STM decline in *Dh31R*, a mutant strain lacking the diuretic hormone 31 receptor, which exhibits compromised learning and memory.

**Conclusion:**

These results demonstrate that *in situ* production of spermidine by a synthetic biotic yeast in the GI tract can enhance STM and further suggest a mechanism involving the gut-brain axis.

## Introduction

The polyamines putrescine, spermidine, and spermine, are produced in all organisms from bacteria to humans. In eukaryotic cells, ornithine decarboxylase (ODC) converts L-ornithine to the diamine putrescine. To synthesize the triamine spermidine, two additional enzymes are required. S-adenosylmethionine (SAM) decarboxylase (SAMDC) converts SAM to decarboxylated SAM (dcSAM). Spermidine synthase transfers an aminopropyl group from dcSAM to putrescine to form spermidine and 5’-methylthioadenosine (MTA). Similarly, spermine synthase transfers an aminopropyl group from dcSAM to spermidine to form the tetraamine spermine and MTA (Pegg, 2016). Polyamines are essential metabolites since deletion of the *Odc* gene causes peri-implantation embryonic lethality in mice (Pendeville et al., 2001). It has been reported that the level of polyamines, especially spermidine, decreases in many mammalian tissues during aging (Nishimura et al., 2006). Recent attention has been focused on the potential of spermidine supplementation as an anti-aging strategy, through extension of lifespan in animal models and amelioration of inflammatory bowel disease, colon cancer, cardiovascular disease, and neurodegeneration (Eisenberg et al., 2009; Madeo et al., 2018). Specifically, free spermidine supplementation in *Drosophila* food extends lifespan (Eisenberg et al., 2009) and prevents aging-related olfactory learning and memory decline (Gupta et al., 2013).

Various molecular mechanisms have been proposed to explain the health benefits of spermidine supplementation. Biochemically, spermidine serves as a precursor for hypusine, a functionally necessary lysine adduct only found in eIF5A (Park et al., 1981), a translation elongation factor essential for overcoming ribosome stalling at polyproline sequence stretches (Pegg, 2016). Hypusination of eIF5A has been proposed to be responsible for spermidine effects on B cell rejuvenation in aging (Zhang et al., 2019), reducing gut inflammation (Gobert et al., 2023), fasting-mediated autophagy and longevity (Hofer et al., 2024) and reducing brain aging in *Drosophila* (Liang et al., 2021). It has also been proposed that spermidine activates mitochondrial trifunctional enzyme in T cells to boost anti-tumor immunity (Al-Habsi et al., 2022) and/or inhibits T cell tyrosine phosphatase to reduce gut inflammation (Mattila et al., 2010; Morón et al., 2013).

The reported diverse health benefits of spermidine supplementation are often at doses that do not significantly change intracellular spermidine levels in target organs, suggesting that spermidine might have a common site of action, namely the gastrointestinal (GI) tract. The GI tract houses the body’s largest immune (Mazziotta et al., 2023) and endocrine (El-Salhy et al., 2017) systems. Spermidine may exert systemic effects by protecting the intestinal epithelium (Penrose et al., 2013) and modulating the immune and endocrine systems. However, as long-term spermidine supplementation has been associated with elevated cancer and stroke risk (Zheng et al., 2022; Park and Igarashi, 2013; Hibino et al., 2023), systemic administration may not be advisable. To avoid this issue, we sought to directly deliver spermidine to the GI tract with minimum effects on systemic spermidine levels. To this end, we have engineered the probiotic yeast *Saccharomyces boulardii* (*Sb*) to overproduce and secrete spermidine. This *Sb* strain, called Sb576, harbors a deletion of the *OAZ1* gene, which encodes Odc antizyme, an inhibitor of Spe1 (Odc1 in yeast) and overexpresses *SPE1*, *SPE2* (which encodes yeast Samdc) and TPO5 (which encodes a pH-independent yeast polyamine exporter). We recently showed that Sb576 retains viability in the feces when administered orally to mice, increases GI lumen spermidine levels, and ameliorates ulcerative colitis and colon cancer in mice (Mohaqiq et al., 2025). In this study, we tested the impact of food supplementation of Sb576 on olfactory learning and memory in *Drosophila*. We found that supplementation of Sb576, but not wild-type *Sb* (SbWT) or free spermidine (1 mM) reduced aging-related learning and memory decline. Moreover, we found that short-term (3 days) supplementation of Sb576, but not SbWT or spermidine, enhanced learning and memory in both young flies and aged flies. Finally, we demonstrated that Sb576 supplementation also reduced aging-associated learning and memory decline in *Dh31R*, a mutant strain lacking the diuretic hormone 31 receptor thought to be involved in learning and memory in *Drosophila*. The results suggest that *in situ* biosynthesis and secretion of spermidine in the GI tract by synthetic biotic yeast can mitigate age-associated decline in learning and memory.

## Materials and methods

### Food preparation and *Drosophila* rearing

Flies were reared in standard *Drosophila* cornmeal food (CM; recipe for 1 L in water: agar, 6.7 g; dry baker’s yeast, 23.1 g; NaKT or potassium sodium tartrate tetrahydrate, 6.30 g; CaCl_2_, 0.53 g; sucrose, 22.8 g; dextrose, 45.5 g; cornmeal, 55.1 g; 8.5 mL of acid mix containing 41.8% proprionic acid and 4.2% of phosphoric acid). Food components and water, minus the acid mixture, were added to a rice cooker, and the mixture was brought to boiling with stirring to dissolve components and then kept at boiling for 20 min. After the stock had cooled down to 60 °C–65 °C, the proprionic acid and phosphoric acid mixture was added and mixed thoroughly. For spermidine supplementation, spermidine trihydrochloride (Sigma Aldrich; made as a 1 M stock in water and stored at −20 °C in single-use aliquots) was added to CM to a final concentration of 1 mM and mixed thoroughly before pouring into bottles. For live yeast supplementation, we prepared CM for 30-day supplementation experiments or CM without dry yeast (CM^–Sc^) for 3-day experiments, as indicated in figure legends.

Live yeast [*Saccharomyces* cerevisiae or ScWT (Breitkreutz et al., 2001), *S. boulardii* strain MYA-796 from ATCC or SbWT, and the spermidine-secreting strain Sb576 (Mohaqiq et al., 2025)] were grown to saturation in rich YPD medium, added to a final density of 5 × 10^7^ cells per mL CM or CM^–Sc^ (at ∼50 °C), and mixed thoroughly before pouring into bottles. We confirmed that the brief exposure to high temperature did not significantly affect yeast viability since live yeast-supplemented food extracts and equivalent amount of input live yeast produced similar number of yeast colony-forming units (CFU). We also found that 5 × 10^7^ per mL was the maximum live yeast permitted in our protocol as higher levels of live yeast supplementation in solid corn meal food resulted in excessive fermentation during incubation at 25 °C causing food cake shrinkage and liquid accumulation in the bottles, trapping and killing the flies. No significant changes in live yeast numbers during incubation at 25 °C, as expected since live yeast were imbedded in the anaerobic food cake.

To obtain sufficient synchronized flies for a typical experiment, we set up multiple bottles each containing 200–300 flies for egg laying. After an overnight incubation, the flies were removed, and the egg-containing bottles were incubated until eclosion. We collected and pooled adult flies, both sexes, emerged within a 24 h period, designated as day 0, and randomly allocated them to control food and the various supplementation groups, 250–300 per bottles. Flies were transferred to fresh food bottles every 3–4 days for long term incubation. All flies were stored in an incubator at 25 °C, with humidity at ∼50% and a 12:12 h light: dark cycle.

### Olfactory learning and memory assays

Short-term memory assays were carried out in a small room set at 25 °C, 60% relative humidity, and under dim red light. Flies were placed into the room 24 h prior to the test to acclimatize. The Fly Training Machine System was purchased from CelExplorer.^1^ Flies (50–100 flies per group) were placed into the training tube and exposed to fresh air (at 2.0 L per min flow rate) for 90 s, followed by exposure to one odor [conditioned stimulus, CS^+^, 0.15% 3-octanol (Sigma Aldrich) in mineral oil or 0.12% 4-methylcyclohexanol (Sigma Aldrich)] in mineral oil simultaneously with electric shock (60 V/1.5 s with 3 s intervals for a total of 60 s). Flies were then given 30 s to rest before being exposed to the second odor (CS^–^, 4-methylcyclohexanol or 3-octanol) without shock for 60 s. Following a 30 s pause, flies were moved to the choice chamber where they were exposed simultaneously to the two odors (CS^+^ and CS), on each side of the chamber, and allowed 120 s to choose a side. Flies were trapped in their respective choice tubes. To count the number of flies in a choice tube, we transferred the flies from the choice tube to a clean polystyrene tube. The tube was gently tapped to force the flies to the bottom before they climbed. Cellphone images were taken when the flies were well spread out in the tube. Counting was done on the cellphone images while marking (using the pen function of photo editing) individual flies to ensure no flies were missed or double-counted. The final numbers also included occasional flies that escaped during transfer operations. Performance index was calculated as the number of flies avoiding the shocked odor (CS^+^) minus the number of flies avoiding the non-shocked odor (CS) divided by the total number of flies in the test and multiplied by 100 (Malik and Hodge, 2014). For each PI value, two groups of flies were tested, one using 3-octanol as CS^+^ and the other using 4-methylcyclohexinol as CS^+^, and the average of the two sub-PIs was taken as PI (*n* = 1). The odor concentrations were chosen based on the results of numerous empirical tests. We ensured that (1) the odors were not significantly aversive when paired with pure air such that during training, the only negative experience should be the electric shock, in a manner that is not compounded by aversive odors; and (2) the two odors were balanced such that untrained flies did not exhibit avoidance toward either odor but trained flies exhibited balanced half PIs.

### Locomotive activity test (climbing assay)

The same flies used for STM assays were used subsequently for the climbing assay on the same day. The two groups of flies for *n* = 1 in the STM test were tested separately in climbing assays, resulting in twice the sample size (*n*). Flies were transferred into a *Drosophila* culture vial with a line marked 6 cm from the bottom, gently tapped the flies down to the bottom, and video-captured while they climbed up the side of the tube. Video was paused at the 10 s-time frame. Flies below and above the 6 cm line were counted from the captured image at the 10 s-time frame and presented as% above the 6 cm line. Each group of flies were tested once with no technical replicates.

### Olfactory acuity

Odor avoidance responses to 3-octanol and 4-methylcyclohexanol was measured by giving a group of untrained flies the option to choose between 1.5% 3-octanol, or 2% 4-methylcyclohexanol, vs. fresh air (all at 2.0 L/min). After 120 s, flies were trapped in their respective choice tubes and counted. Performance index was calculated as the number of flies collected in fresh air arm minus that in the odor arm, divided by total number and multiplied by 100 (Malik and Hodge, 2014). Like odor concentrations used in the learning and memory assays above, odor concentrations for the odor acuity tests were also determined empirically as the minimum concentrations that, when paired with pure air, elicited the maximum aversive behavior in untrained control flies.

### Shock reactivity

Shock reactivity was quantified by placing a group of untrained flies into the T-maze where one side of the maze had a training tube where the shock (60 V/1.5 s with 3.5 s intervals for a total of 60 s) was delivered. The other side of the maze had the tube but not shock. After 1 min, flies were trapped into their respective tubes and counted. The performance index was calculated as number of flies recovered from the non-shock tube minus that from the shock tube, divided by the total number and multiplied by 100 (Malik and Hodge, 2014).

### Polyamine determination

Polyamines were extracted from whole flies according to Qin et al. (2021) with slight modifications. Briefly, 30 flies (15 male and 15 female) were frozen in liquid N_2_ and ground using a disposable pestle. Boiling water (495 μL) was mixed with 5 μL of 100 μg/mL deuterated spermidine, Spermidine-(butyl-d_8_) trihydrochloride (709891; Sigma-Aldrich), and the mixture was added to each ground sample. The tube was then vortexed vigorously to suspend the ground sample and kept boiling for 30 min. Tubes were then placed on ice for 5 min. The post centrifugation (twice at 15,000 rpm at 4 °C,15 min) supernatant was then stored at −80 °C or directly subjected to the quantification step. All extracted samples were analyzed by using a validated liquid chromatography mass spectrometry/mass spectrometry (LC-MS/MS) method, as described in our previous publication (Mohaqiq et al., 2025).

### RNAseq

To eliminate/reduce yeast (and yeast RNA) in the intestine, flies were transferred to bottles containing filter paper saturated with 5% glucose in water for 4 h. The flies were anesthetized by CO_2_ and the entire intestine excluding the crop was excised in PBS. Isolated guts were then placed into Eppendorf tubes. Total RNA was isolated from 30 female guts using Qiagen’s RNeasy Plus kit. RNA sample quality assessment was performed with the Fragment Analyzer Standard Sensitivity RNA assay (Agilent) and concentration measured with the Qubit 3.0 HS RNA assay (Thermo). NGS libraries were prepared with the Stranded mRNA library prep kit (Illumina) using 400 ng of total RNA input. Sequencing was performed with the Nextseq 2,000 P1 100 cycle flow cell (Illumina). FASTQ sequencing file reads were aligned to the *D. melanogaster* genome BDGP6.46 genome, Ensembl transcriptome 59 using the nf-core/rnaseq pipeline (Ewels et al., 2020) version 3.14.0. Since no biological replicates were performed, fold change estimates between samples were generated using GFOLD (Feng et al., 2012) to compare STAR/salmon pseudocount values.

### Statistical analysis

Statistical analysis and graphing were performed using GraphPad Prism software. All quantitative values are shown as means ± standard error of the mean (SEM). To compare multiple group means, one-way ANOVA analysis was used followed by Tukey’s multiple comparisons test. To compare two group means (Figure 5), *t*-test (two tailed) was used. *, *p* < 0.05; **, *p* < 0.01; ***, *p* < 0.001; ****, *p* < 0.0001.

## Results

### Long-term supplementation of Sb576 reduces aging-associated learning and memory decline

**FIGURE 1:**
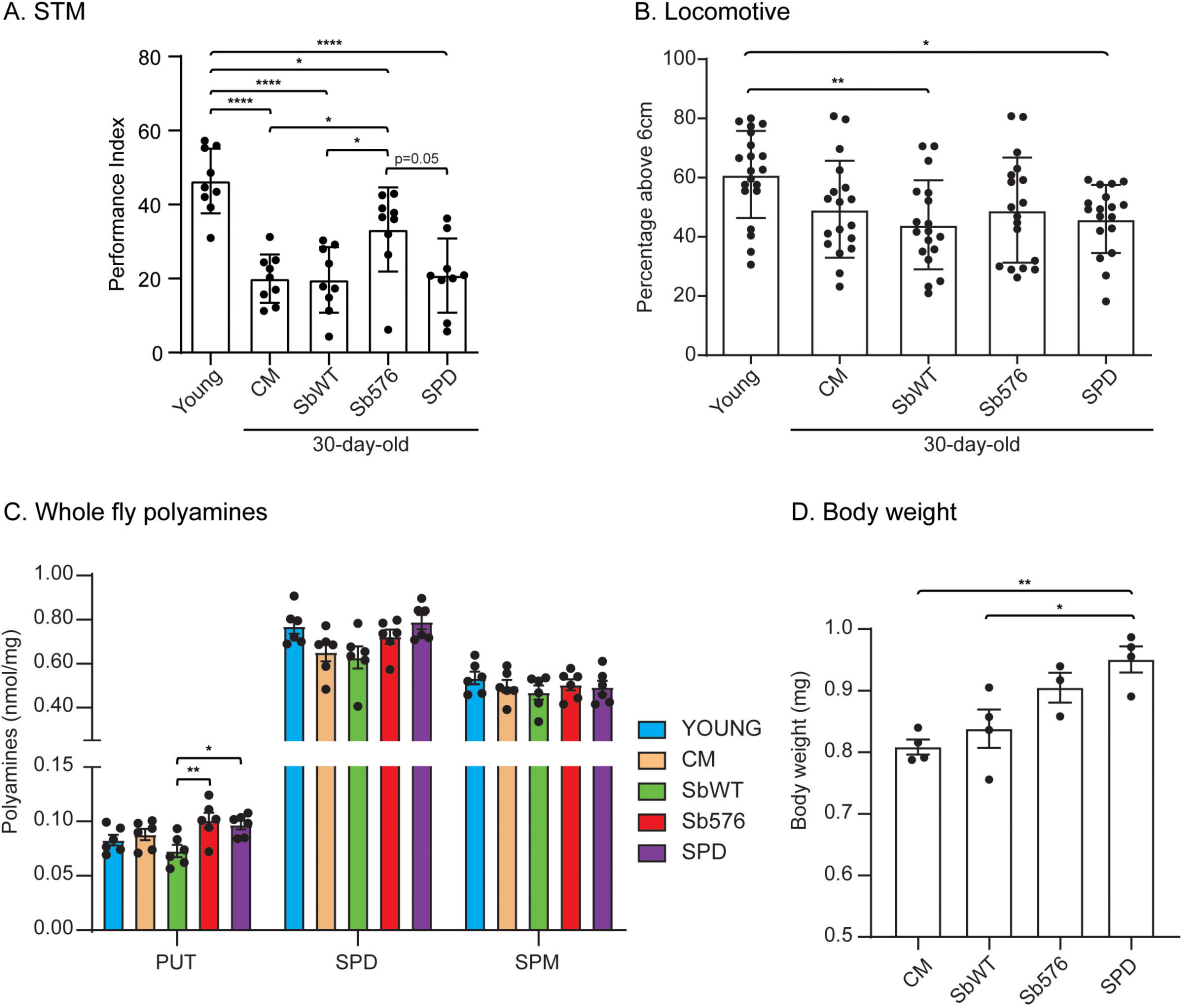
Sb576 reduces aging-associated short-term memory (STM) decline in flies. **(A)** STM performance scores of young flies and flies after 30-day feeding on CM without supplementation or supplemented with spermidine or live wild-type *Sb* SbWT or Sb576. **(B)** Locomotive ability test of the same flies following the olfactory memory test in **(A)**. **(C)** Whole body polyamine levels in young flies and 30-day old flies fed on CM or CM plus the indicated supplementation. **(D)** Body weight of 30-day-old flies after feeding on CM or CM supplemented with spermidine or live SbWT or live Sb576. Each dot represents the mean of a group of flies (between 58 and 91, both male and female). **(A–D)** Shown are means with SEM. One-way ANOVA and Tukey’s multiple comparisons test. *, *p* < 0.05; **, *p* < 0.001; ****, *p* < 0.0001.

To explore the efficacy of the spermidine secreting strain Sb576 in delaying aging-related memory decline, we employed the *D. melanogaster* olfactory learning and memory model (Gupta et al., 2013). We employed w^1118^(isoCJ1) strain (provided by Dr. Joshua Dubnau of Stonybrook), which is derived from outcrossing w^1118^ (white mutant with white eyes) into Canton-S wild-type strain followed by repeated backcrossing to Canton-S wild-type strain to obtain an isogenic wild-type with w^1118^ (white eyes) (Yin et al., 1995), referred to as wild-type (w^1118^) or WT (w^1118^) thereafter. Newly eclosed flies were fed on fly food (cornmeal, CM, containing cooked baker’s yeast; see section “Materials and methods” for details) supplemented with either live SbWT, or the engineered spermidine-secreting strain Sb576, or with 1 mM spermidine (see section “Materials and methods for details) for 30 days. Thirty-day-old flies were subjected to an olfactory learning and memory test according to the protocol of Malik and Hodge (2014) with modifications described in Materials and methods, using young flies (3-day-old) as a control. As expected, we found that 30-day-old flies fed on CM food had significantly lower performance index (PI) compared to young flies (Figure 1A, young vs. CM). Supplementation of CM with SbWT or with spermidine did not impact PI. However, Sb576 supplementation significantly improved PI, not only compared to CM-fed flies, but also compared to SbWT-supplemented flies and, to a slightly lesser degree (*p* = 0.05), to spermidine-supplemented flies (Figure 1A). We also subjected the flies to locomotive ability testing and found that in general young flies performed better than 30-day-old flies, also as expected. However, there was no significant difference in locomotive ability amongst the 30-day-old flies regardless of the food supplementation regime (Figure 1B). We extracted polyamines from whole flies and performed LC-MS/MS analyses and quantification (Figure 1C). These data revealed significant differences in spermidine levels amongst the groups (*p* = 0.027, one-way ANOVA; no significant difference between any two groups in Tukey multiple comparison test). Specifically, 30-day old flies (CM) had reduced spermidine levels compared to young flies. Spermidine supplementation, and Sb576 supplementation to a lesser degree, restored spermidine levels. In contrast, there was no significant difference in spermine (SPM) levels in any of the groups. Supplementation with Sb576 and with spermidine also significantly increased putrescine (PUT) levels compared to SbWT-supplemented group, although the overall putrescine levels were low and there was no difference between young and old flies (young vs. CM). The increase in putrescine levels in flies supplemented with spermidine has been observed previously (Eisenberg et al., 2009), indicating that catabolic conversion of spermidine to putrescine (Gerner and Meyskens, 2004) is active in flies. As a surrogate for food intake, we also measured body weight of flies after 30-day food supplementation. Interestingly, 30-day-old flies after spermidine supplementation were significantly heavier than 30-day-old flies fed with CM food or CM food supplemented with SbWT (Figure 1D), consistent with the roles of polyamines in growth (Pegg, 2016). Together, these results suggest that Sb576 supplementation reduces aging-associated STM decline without affecting aging-associated locomotive decline in *Drosophila*. We note that despite restoring spermidine levels in 30-day old flies to those found in young flies (Figure 1C), free spermidine supplementation failed to reduce aging-associated STM decline (Figure 1A), in contrast to a previous report (Gupta et al., 2013).

### Short-term Sb576 supplementation enhances STM in young and old flies

**FIGURE 2:**
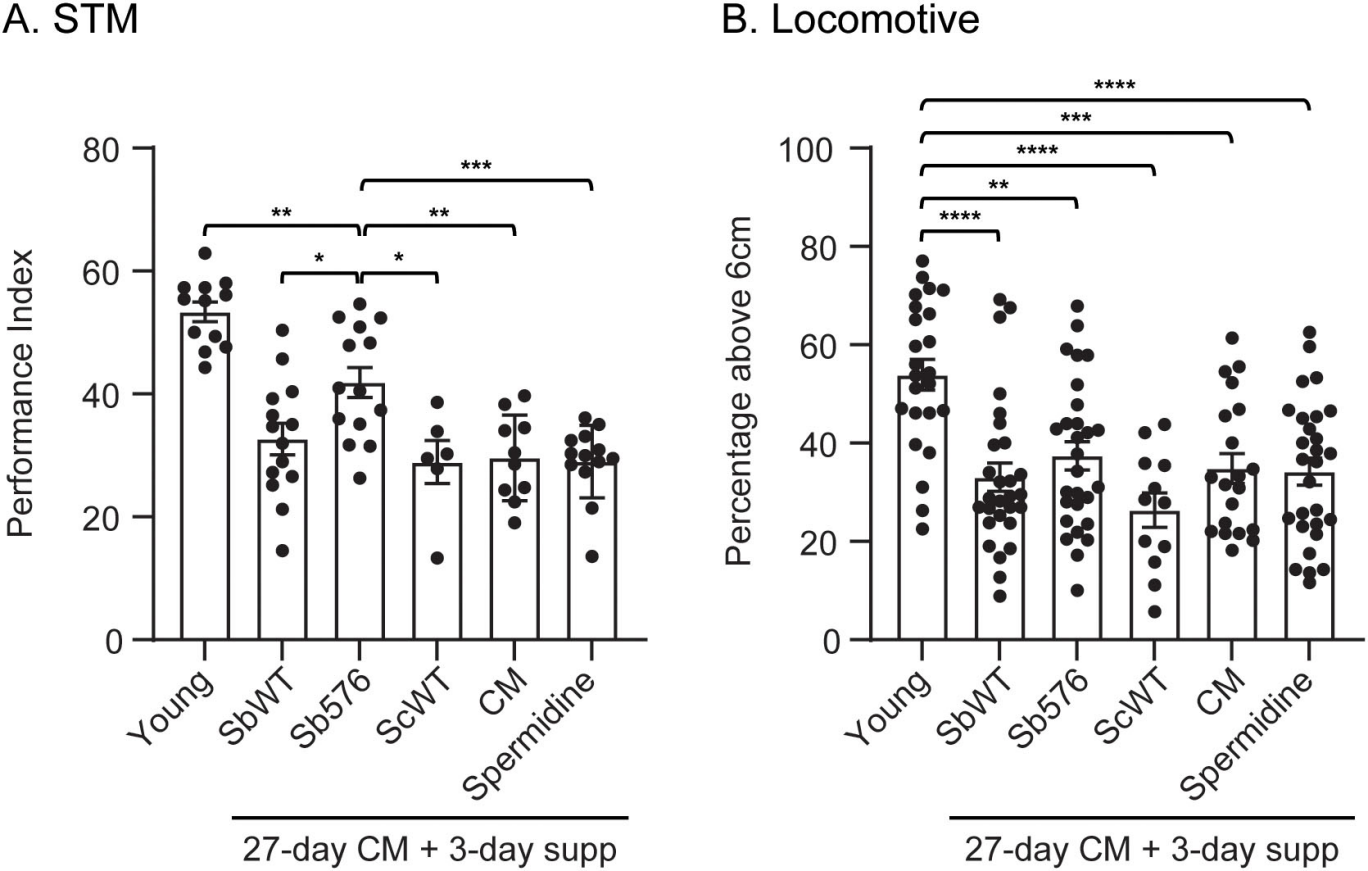
Sb576 enhances short-term memory (STM) in aged flies. Newly eclosed flies were fed on cornmeal food (CM) for 27 days before transferring to yeast-free cornmeal food (CM^–Sc^) supplemented with live ScWT, SbWT, or Sb576, or to cornmeal food (CM) with or without 1 mM spermidine. Flies were tested 3 days later, with young flies as control, for olfactory memory **(A)** and locomotive ability **(B)**. Shown are means with standard error of the mean (SEM). In addition to the differences shown, the young group is significantly different (****) from all other groups **(A)**. One-way ANOVA and Tukey’s multiple comparisons test. *, *p* < 0.05; **, *p* < 0.01; ***, *p* < 0.001 ****, *p* < 0.0001.

Given the specific effects of Sb576 on aging-related STM decline, but not on aging-related locomotive decline, we wonder if the Sb576 strain might impact STM independently of aging processes. Specifically, we wanted to know if Sb576 can improve learning and memory in older flies with learning and memory deficit. *D. melanogaster* have an average lifespan of 48 days after eclosion; they exhibit significant decline in STM as early as 10-day of age and reach the lowest point by the age of 30 days (Tamura et al., 2003). We cultured newly eclosed flies for 27 days in regular CM before splitting them in various supplementation groups for additional three days. For the 3-day supplementation experiments, we used CM food without cooked (killed) baker’s yeast (CM^–Sc^) and supplemented with live baker’s yeast (*Saccharomyces cerevisiae*, Sigma 1278b, or ScWT), or CM^–Sc^ supplemented with SbWT or Sb576. We also included groups on CM alone, and CM supplemented with spermidine (1 mM) for the 3-day supplementation. All flies were subjected to the same STM test as 30-day-old flies, with young flies as a control. First, replacing cooked baker’s yeast (at 23.1 g per L, estimated at 2-4 × 10^8^ cells/mL) with live baker’s yeast (at 0.5 × 10^8^ cells/mL) for the last three days did not affect performance in olfactory learning and memory assays (Figure 2A, ScWT vs. CM). Second, there was not significant difference between ScWT and SbWT, indicating that probiotic alone did not improve memory performance. Third, and most importantly, 27-day-old flies treated with live Sb576 supplementation for three additional days performed significantly better than all other 30-day-old groups. Despite the improvement in STM of old flies fed Sb576, we still observed a significant difference in STM performance between the Sb576 group and young flies (Figure 2A), indicating that the 3-day treatment with Sb576 only partially rescued the memory deficit lost during aging. In contrast to STM performance, Sb576 supplementation for three days did not significantly alter locomotive activity in old flies (Figure 2B).

**FIGURE 3:**
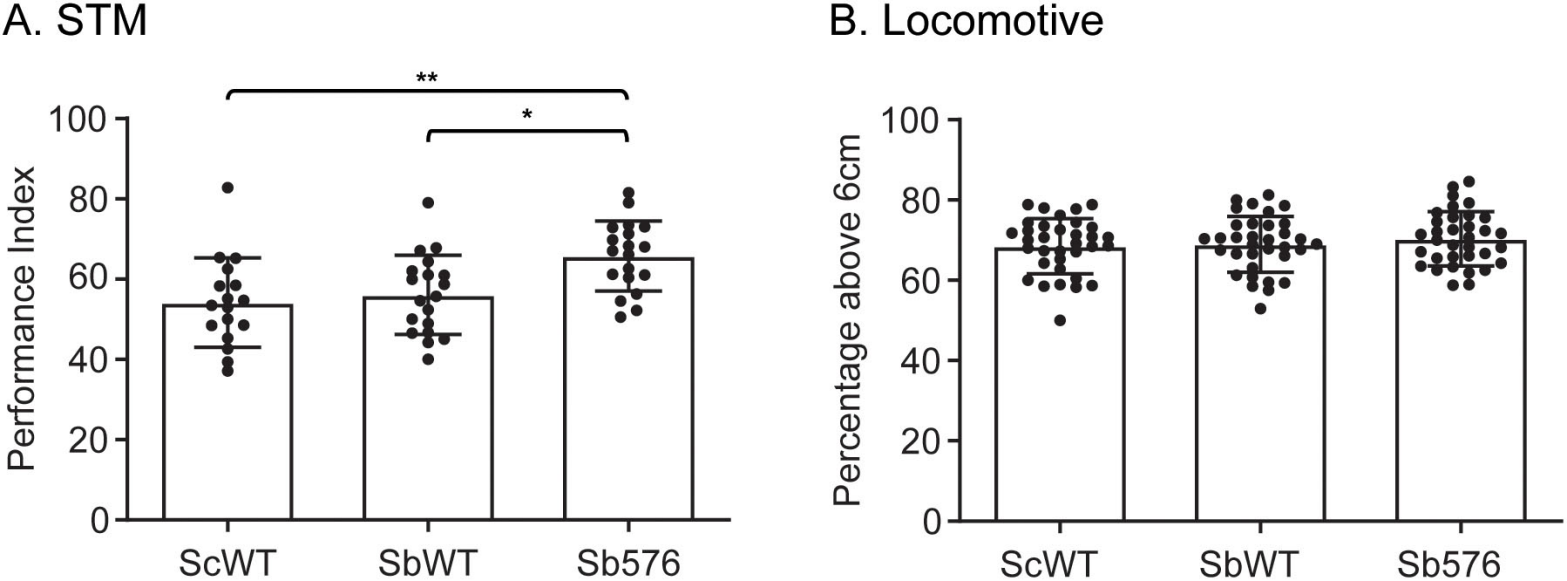
Sb576 enhances short-term memory (STM) in young flies. Newly eclosed flies after three-day feeding on CM^–Sc^ supplemented with live ScWT, SbWT, or Sb576 were tested for olfactory memory (A) and locomotive ability (B). Shown are means with SEM. One-way ANOVA and Tukey’s multiple comparisons test. *, *p* < 0.05; **, *p* < 0.01.

To further establish the memory enhancing effects of Sb576, we tested the various yeast supplementation on young flies. Newly eclosed flies were transferred to CM^–*Sc*^ with the indicated live yeast supplementation and cultured for three days followed by STM test. We found that Sb576 supplementation significantly improved STM performance compared to supplementation with either ScWT or SbWT (Figure 3A). No difference was found in any group in a locomotion test (Figure 3B). To rule out the possibility that Sb576 may alter odor perception or response to electric shock used in conditioning and testing for STM assays, we performed odor acuity and shock reactivity tests and found no difference among the three groups of flies (Table 1).

**TABLE 1:** Odor acuity and shock reactivity.

	MCH	OCT	Shock reactivity
**Olfactory avoidance**			
ScWT	62.9 ± 2.5	83.4 ± 8.2	82.6 ± 4.4
SbWT	66.8 ± 8.4	84.6 ± 3.5	71.9 ± 6.8
Sb576	67.5 ± 9.8	80.0 ± 7.7	67.7 ± 13.4

Performance index (means with SEM, *n* = 3) of WT (w^1118^) flies fed on food supplemented with the indicated live yeast. Newly eclosed flies were fed for 3 days before testing for odor acuity or 4 days before testing for electric shock reactivity.

Taken together, our results suggest that live Sb576 supplementation reduced aging associated learning and memory decline and enhanced learning and memory performance in young flies, as well as in old flies with learning and memory deficit.

### Sb576 significantly reduced aging-associated memory decline in Dh31R flies

**FIGURE 4:**
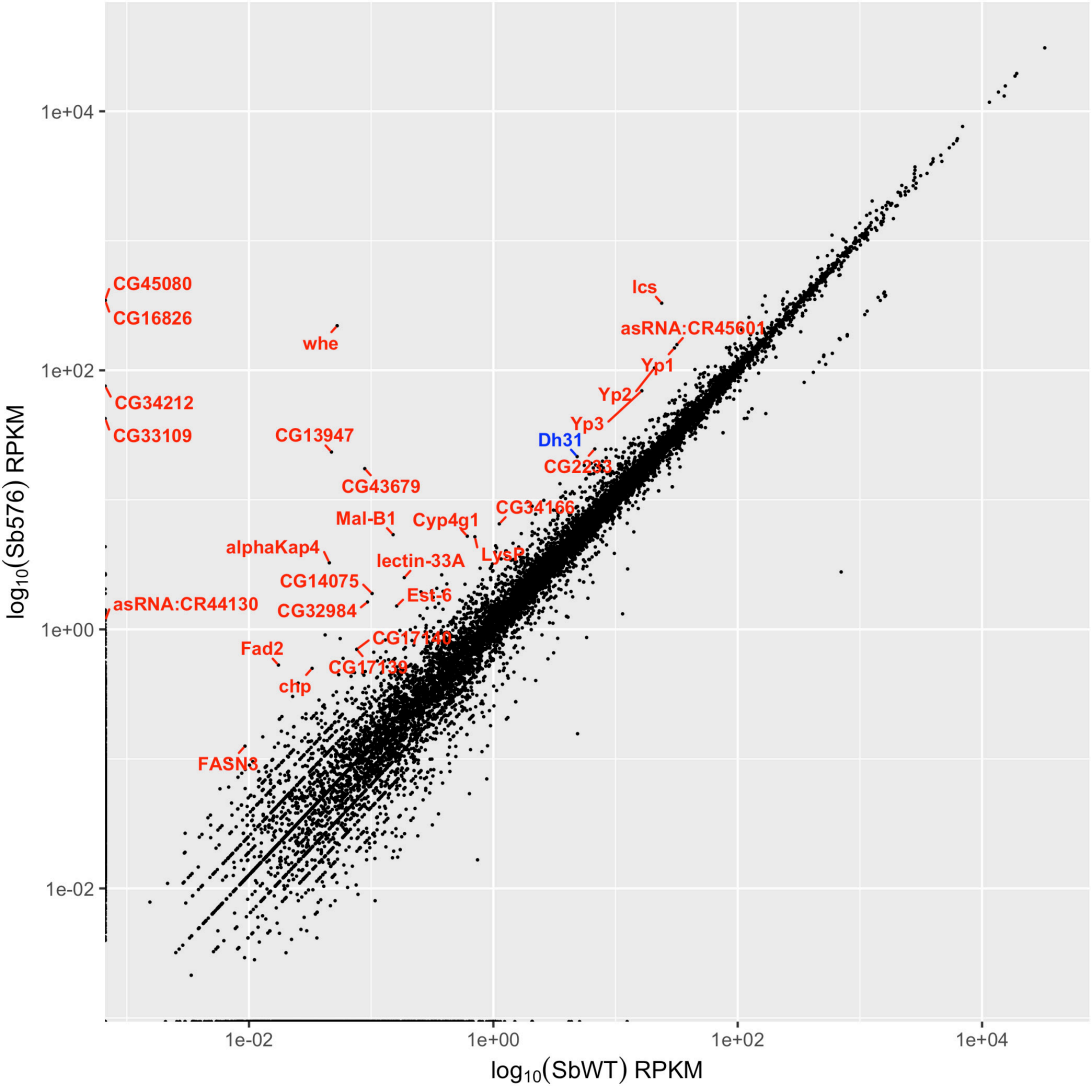
Top ranked overexpressed intestinal genes in Sb576 group compared to SbWT group. Only genes with GFOLD greater than 1.5 are labeled. Dh31 is highlighted in blue.

**FIGURE 5:**
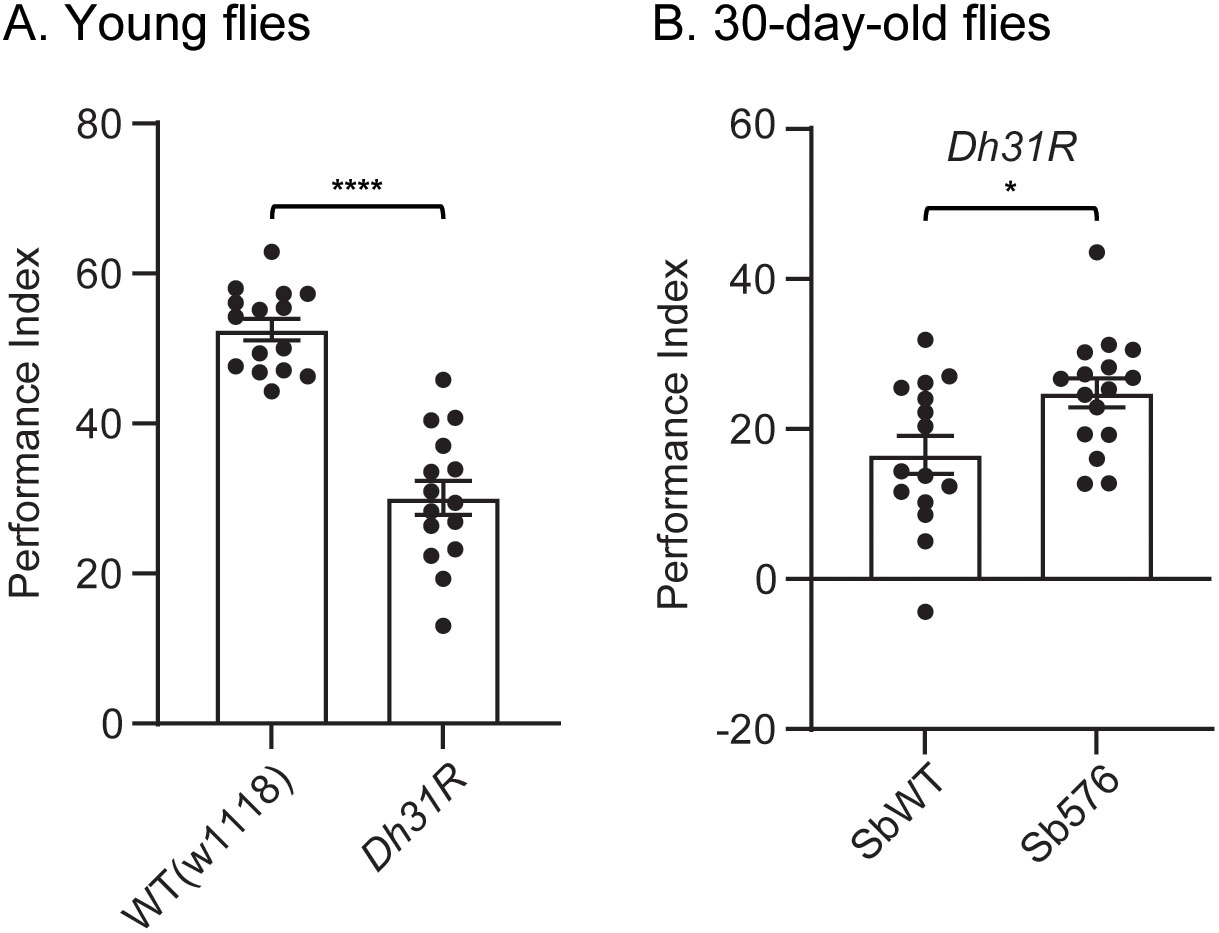
Sb576 reduces aging-associated short-term memory (STM) decline in *Dh31R* flies. **(A)** Performance index of young WT (w^1118^) flies compared with young *Dh31R* flies. **(B)** Performance index of 30-day-old *Dh31R* flies fed on CM food supplemented with live SbWT or Sb576. Two-tailed *t*-test. *, *p* < 0.05; ****, *p* < 0.0001.

The impact of short-term 3-day Sb576 supplementation on STM suggested a potential direct mechanism of action involving the gut brain axis. To explore this possibility, we carried out RNAseq analysis of intestines from young versus SbWT- or Sb576-treated old flies (i.e., those shown in Figure 1). Whereas numerous differentially expressed genes were found between young flies and the two groups of old flies, few such differentially expressed genes were found between SbWT- and Sb576-treated old flies (see Supplementary Tables 1–3 for details). Specifically, only 29 genes with GFOLD values (Feng et al., 2012) greater than 1.5 were found over-expressed in Sb576-treated group compared to SbWT-treated group (Figure 4). All three yolk protein genes (Yp1, Yp2, and Yp3) were among this group, suggesting a positive impact of Sb576 administration on egg production and fecundity (Tarone et al., 2012). One neuropeptide gene, the diuretic hormone Dh31 was also amongst the 29 differentially overexpressed genes (Figure 4). Interestingly, it has been recently reported that Dh31 signaling is involved in olfactory learning and memory in *D. melanogaster* (Lyu et al., 2023). We therefore decided to test if Sb576 supplementation can reduce aging-associated learning and memory decline in Dh31 receptor null mutant flies, *Dh31R* (Lin et al., 2022) (provided by Dr. Jing Wang of University of California, San Diego). We first compared STM performance of young WT (w^1118^) flies vs. young *Dh31R* flies (Figure 5A), confirming that *DH31R* flies had significantly lower performance index comparted to WT (w^1118^) flies (Lyu et al., 2023). We then fed newly eclosed *Dh31R* mutant flies with SbWT- or Sb576-supplemented food for 30 days followed by subjecting the flies to olfactory learning and memory assays. Our data indicated that Sb576 still significantly reduced aging-associated memory decline in *Dh31R* flies (Figure 5B), suggesting that the effects of Sb576 supplementation on learning and memory is not fully dependent on Dh31 signaling.

## Discussion

We have demonstrated that supplementation of *D. melanogaster* food with the engineered spermidine-secreting *Sb* strain Sb576 reduced aging-related decline in learning and memory (STM). Moreover, we have shown that short-term (3 days) Sb576 supplementation enhanced STM in young flies, as well as in aged flies with memory deficit. This memory enhancement was unique to Sb576 and not shared by supplementation with either the SbWT control strain or free spermidine (1 mM). Furthermore, enhancement of learning and memory by Sb576 is not accompanied by enhancement of locomotive ability, suggesting that dietary Sb576 supplementation specifically influences brain functions. Since we performed climbing assays on the same flies following STM assays on the same day, we cannot rule out the possibility that the prior STM assays may have affected the outcome of the subsequent climbing assays, potentially obscuring subtle differences.

The inability of spermidine supplementation to reduce aging-associated memory decline contrasted with previous finding that spermidine supplementation (1 or 5 mM) reduced aging-associated decline of both STM and intermediate term memory (ITM) (Gupta et al., 2013). Although the reason for this apparent discrepancy is not known, we note that in previous studies spermidine supplementation was initiated during parental fly mating and then continued through egg-to-eclosion development and aging (Gupta et al., 2013, 2016; Liang et al., 2021), in contrast to our live yeast or spermidine supplementation which commenced after eclosion. It is possible that the beneficial effects of free spermidine supplementation reported previously require prolonged administration over the entire lifespan of the animal, starting from fertilized eggs (Gupta et al., 2013; Liang et al., 2021). We note that our spermidine supplementation regime was sufficient to restore spermidine levels in old flies to the level found in young flies at least as efficiently as Sb576. In fact, spermidine supplementation, but not Sb576 supplementation, significantly increased body weight of 30-day-old flies, suggesting that systemic spermidine supplementation stimulates growth (Pegg, 2016). Thus, the inability of direct spermidine supplementation to reduce memory decline in aged flies in our study was unlikely to be due to inadequate spermidine supplementation.

Polyamines, especially spermidine and spermine, are potent enhancers of *N*-methyl-D-aspartate (NMDA) receptor signaling through potentiation of ligand binding to the receptor (Romano et al., 1991). However, the effect of exogenous spermidine supplementation on learning and memory may be complex. Long-term oral spermidine supplementation improves brain functions in flies (Gupta et al., 2013, 2016; Liang et al., 2021) and mice (Schroeder et al., 2021; Maglione et al., 2019). Since spermidine has very limited permeability through the blood-brain barrier (Shin et al., 1985; Diler et al., 2002), the effects of spermidine on brain functions in these long-term supplementation experiments may be indirect. Consistent with this interpretation, spermidine levels have been found to decline in some mouse tissues but not in the brain during aging (Nishimura et al., 2006). Indeed, Rushaidhi et al. (2012) found that spermidine levels increase in some hippocampal subregions and do not change in others during rat aging. Short-term intrahippocampal infusion of spermidine after training improves brain functions in rats, likely through direct modulation of NMDA receptors in postsynaptic neurons (Fabbrin et al., 2020; Signor et al., 2016; Berlese et al., 2005). In addition, intracerebroventricular injection of drugs that inhibit spermidine synthesis or disrupt spermidine-NMDA receptor binding counteracts β Amyloid peptide-induced memory impairment in mice, likely through modulating extrasynaptic NMDA receptors (Gomes et al., 2014).

Since free spermidine was as effective as Sb576 in restoring spermidine levels in aged flies yet did not impact STM in flies, it seems unlikely that the effects of Sb576 on STM occur through direct interaction of Sb576-derived spermidine with brain NMDA receptors. One possibility is that dietary spermidine and spermidine derived from Sb576 interact with different regions/cells of the fly intestine. For example, dietary or free polyamines are predominantly absorbed in the small intestine, especially in the duodenum and proximal jejunum, with little directly absorbed in the large intestine in rats and mice (Milovic, 2001; Nakamura et al., 2021). In contrast, orally administered Sb576 remains viable and elevates luminal spermidine levels in the large intestine in mice (Mohaqiq et al., 2025). Alternatively, it is formally possible that engineering of the polyamine synthetic and transport pathways may have indirectly altered the probiotic properties of *S. boulardii* to enhance learning and memory independently of spermidine secretion. Regardless, we propose that Sb576 enhances memory in flies through a mechanism mediated by the gut-brain axis. One example of gut-brain signaling is the nutritional (specifically via amino acids) stimulation of production of the gut hormone Dh31 to control mating behavior in *D. melanogaster* (Lin et al., 2022). Although Sb576 enhancement of learning and memory in flies does not depend on Dh31 signaling, these experiments underscored the remarkable ability of Sb576 to reduce learning and memory decline even in strains that lack Dh31R. Further work will be required to uncover the mechanisms by which *in situ* production of spermidine in the GI tract enhances learning and memory in *Drosophila*.

## Data Availability

The data presented in the study are deposited in the ArrayExpress repository, accession number E-MTAB-15806, available at: https://www.ebi.ac.uk/biostudies/arrayexpress/studies/E-MTAB-15806.

## Footnotes

1
https://www.celexplorer.com/product_list.asp

## Supplementary material

Supplementary Table 1 GFOLD - Young vs. Old_Sb576.

Supplementary Table 2 GFOLD - Young vs. SbWT.

Supplementary Table 3 GFOLD - SbWT vs. Sb576.
